# PReS-FINAL-2225: Oxidative stress in children with episodic fever of unknown origin

**DOI:** 10.1186/1546-0096-11-S2-P215

**Published:** 2013-12-05

**Authors:** J Vojinovic, J Radovic, D Lazarevic, T Jeftic-Stoimenov, D Pavlovic

**Affiliations:** 1 Department of Pediatric Rheumatology, Clinical Center, School of Medicine, University of Nis, Nis, Serbia; 2 Institute of Pathophysiology, University of Nis, Nis, Serbia; 3 Institute of Biochemistry, Faculty of Medicine, University of Nis, Nis, Serbia

## Introduction

Fever of unknown origin (FOU) i.e.: (1) a temperature greater than 38.3°C on several occasions, (2) more than 3 weeks' duration of illness, and (3) failure to reach a diagnosis despite 1 week of inpatient investigation are commonly seen in pediatric practice. The conditions could be caused by mutations of genes coding inflammasome sequences. In these instances activated neutrophils and monocytes intensively generate reactive oxidative species.

## Objectives

Aim of this study was to evaluate if there are oxidative changes of lipids in plasma and erythrocytes and advanced oxidation protein products (AOPP) in children with episodic FUO.

## Methods

The study enrolled 25 children with episodic FUO (in afebrile phase) and 25 healthy age matched controls. Lipid peroxidation was evaluated measuring malondialdehyide (MDA) production by thiobarbituric-acid-reactive substances (TBARS) assay in plasma and erythrocytes while advanced oxidation protein products in plasma (AOPP) was measured in plasma using spectrophotometric methods to determinate TBARS and AOPP levels.

## Results

Mean duration of episodic fevers was 3.96 ± 2.8 years. Levels of erythrocytes MDA were higher in patients than in controls (86.26 ± 10.75 vs. 78.0 ± 3.21 nmol/g Hgb), however not significantly. There was no difference in MDA concentrations in plasma (2.42 ± 0.35 vs. 2.41 ± 0.39 μmol/L). Interestingly, levels of AOPP were significantly lower in patient group than in controls (18.8 ± 5.04 vs. 25.1 ± 3.35 μmol/L, p < 0.05).

## Conclusion

The results confirm that erythrocyte membrane is the most vulnerable to the oxidative stress. However, duration of episodic fevers for approximately 4 years is not sufficient to cause significant oxidative modifications of lipids and proteins. Unexpected results for AOPP could be explained through the higher anti-oxidative capacity of blood.

## Disclosure of interest

None declared.

